# TRAF4, at the Crossroad between Morphogenesis and Cancer

**DOI:** 10.3390/cancers3022734

**Published:** 2011-06-21

**Authors:** Adrien Rousseau, Marie-Christine Rio, Fabien Alpy

**Affiliations:** Institut de Génétique et de Biologie Moléculaire et Cellulaire (IGBMC), UMR 7104 CNRS, U964 INSERM, Université de Strasbourg, BP 10142, 67404 Illkirch, C.U. de Strasbourg, France; E-Mails: Adrien.Rousseau@igbmc.fr (A.R.); rio@igbmc.fr (M.-C.R.)

**Keywords:** TRAF, reactive oxygen species, cell polarity, morphogenesis, cancerogenesis, smurf

## Abstract

*Tumor Necrosis Factor Receptor-Associated Factor 4 (TRAF4)* is a gene whose expression is altered in cancers. It is overexpressed in a variety of carcinomas of different origins, often as a consequence of amplification. TRAF4 encodes an adaptor protein that belongs to the TRAF protein family. While most TRAF proteins influence immune and inflammation processes, TRAF4 is mainly involved in developmental and morphogenic processes. Interestingly, this protein has been shown to be linked to crucial cellular functions such as cell polarity and the regulation of reactive oxygen species production.

## 1. Introduction

The understanding of the molecular alterations involved in cancer development must take into account the heterogeneity of the tumors. This is a prerequisite for precise prognosis assessment and ultimately the development of adapted therapeutic strategies. A high level of expression of a subset of genes is a common alteration found in cancers. Indeed, overexpression is one of the mechanisms that leads to the activation of proto-oncogenes to oncogenes [1]. The *Tumor Necrosis Factor (TNF) Receptor Associated Factor 4 (TRAF4)* gene was identified due to its high expression in breast cancers [2,3]. TRAF4 was originally called Metastatic Lymph Node (MLN) 62 and CART1 (C-rich motif associated with RING and TRAF containing protein 1) [3,4]. This gene encodes a member of the TRAF family, a group of scaffold proteins that link TNFR and Toll/IL-1 family members to signaling cascades [5-7]. Recently, the function of TRAF4 was shown to be linked with specific cellular processes such as reactive oxygen species production and cell polarity. In this review, we discuss the recently identified roles of TRAF4 and its essential function during embryonic development.

## 2. TRAF4 belongs to the TRAF Protein Family

**Figure 1.**
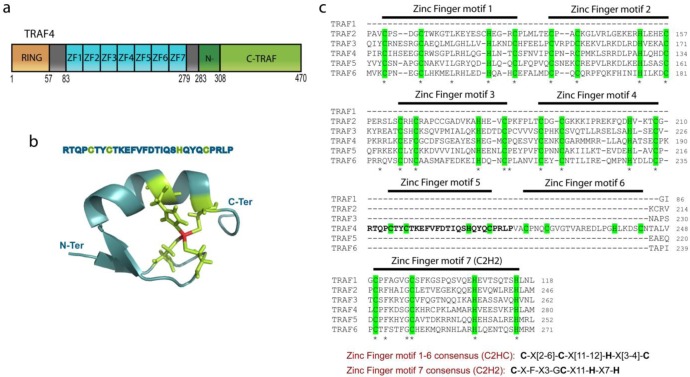
Modular organization of the human TRAF4 protein. **(a)** TRAF4 is a 470 amino acid protein, harboring conserved RING, Zinc Finger (ZF1-ZF7) and TRAF domains. **(b)** Cartoon diagram of the nuclear magnetic resonance (NMR) solution structure of TRAF4 zinc finger motif 5 (PDB ID : 2EOD). Residues interacting with the zinc atom (red) are represented by green sticks. **(c)** Alignment of the zinc finger containing region of TRAF proteins performed with the ClustalX software [27]. Identical residues in all sequences are shown by an asterisk. Amino acids interacting with zinc atoms are highlighted in green. Residues corresponding to the sequence shown in panel b are in bold type.

The TRAF protein family is composed of seven members that share a common structural organization (Figure 1) [2,6,8-10]. All TRAF proteins contain a C-terminal TRAF domain except TRAF7 where this domain is substituted by seven WD40 repeats [11,12]. The TRAF domain, which is involved in the homo- and heterotrimerization of TRAF proteins is mushroom-like in shape with a stalk-like N-TRAF and a cap-like C-TRAF [11,13-16]. The N-TRAF domain forms a trimeric parallel coiled-coil conformation [11]; in TRAF4, this region is short compared with other TRAF family members as it contains only 3 heptads while other TRAF proteins contain more than 10 heptads [4]. This difference might explain the poor ability of TRAF4 to associate with other TRAF proteins [17]. The C-TRAF domain forms an eight-stranded β-sandwich, a fold that is not restricted to TRAF proteins. Indeed, this domain is also known as the meprin and TRAF-C homology (MATH) domain because of its sequence homology with the meprin extracellular metalloprotease family [18-20]. The C-TRAF domain is involved in the trimerization of TRAF proteins. Moreover, it serves as the docking site of upstream partners during signaling by interacting directly with membrane receptors or indirectly with proteins attached to these receptors. For instance, key surface residues of the TRAF2 C-TRAF domain are involved in its interaction with the TNF receptor superfamily member, TNFR2 [14]. The N-TRAF domain is also involved in protein-protein interactions such as in TRAF2 where it is the binding site for the E3-ligase c-IAP2 [21].

All TRAFs, except TRAF1, contain two additional domains, an N-terminal RING (Really Interesting New Gene) finger motif and several successive zinc fingers. The RING domain is characterized by a C3HC4 motif which forms two zinc fingers [22]. However, TRAF4, 5, 6 and 7 contain a C3HC3D motif [4]. The RING domain mediates a crucial step in the ubiquitination pathway by simultaneously binding ubiquitination enzymes and their substrates, hence functioning as an E3-ligase [23]. However, the function of the TRAF4 RING domain as an E3-ligase has not yet been demonstrated. The central part of TRAF4 is formed by a cysteine-rich region that was previously defined as the association of three CART domains [4]. Each CART domain was proposed to be composed of two HC3HC3 zinc fingers. However, the solution NMR structure of the cysteine-rich central region (PDB ID: 2YUC, 2EOD) suggests that this part of TRAF4 is likely composed of 7 successive zinc fingers as previously proposed by several authors [6,24]. The first six zinc fingers have a C2HC structure while the seventh has a C2H2 structure (Figure 1). These data are consistent with the crystal structure of the zinc-finger region of TRAF2 and 6 [25,26].

Unlike other members of the TRAF protein family, TRAF4 does not associate with most members of the TNFR family. For instance, while TRAF1, 2, 3, 5, 6 have been reported to be directly or indirectly recruited to the cytoplasmic tail of CD40 (TNFR superfamily member 5), TRAF4 does not interact with this receptor [28-31]. However, weak interactions between TRAF4 and Lymphotoxin β receptor (LTβR) or Nerve Growth Factor Receptor (NGFR alias p75 neurotrophin receptor) have been reported, although the physiological relevance of these interactions remains poorly understood [32,33]. In agreement with these data, overexpression of TRAF4, unlike other members of the TRAF family, fails to activate NF-κB [34].

The primary structure of TRAF4 suggests that this protein, despite some differences, must share similar functional characteristics with other TRAF proteins. TRAF4 is likely to be involved in signal transduction as its TRAF domain mediates the direct or indirect interaction with a transmembrane receptor. Moreover, TRAF4 may possess E3-ligase activity through its RING domain which could mediate the poly-ubiquitination of target proteins and lead to their activation or degradation. However, the identity of the signaling pathway and the potential molecular targets of its putative E3-ligase activity remain unknown.

## 3. TRAF4 has a Special Place during Evolution

TRAF genes arose early during evolution. This protein family is represented by one member DG17 (alias zfaA) in the social amoeba *Dictyostelium discoideum* where it is expressed during the aggregation step of the life cycle of these unicellular organisms, when individual cells aggregate to form a multicellular colony [4,35]. TRAF family members are also present in Metazoans. One TRAF protein HyTRAF1 has been identified in the hydroid *Hydractinia echinata* [36]. HyTRAF1 is proposed to be involved in the regulation of apoptosis owing to its high expression during larval stages [36]. In *Caenorhabditis elegans*, this family is still represented by a single member named ceTRAF [37]. In *Drosophila*, the TRAF protein family is more diversified with three members, DTRAF1 to DTRAF3. Finally, in mammals 7 members are present and among them TRAF7 has a peculiar place as it is devoid of a TRAF domain. Sequence analysis of the TRAF family members in these different species suggests that human TRAF4 and TRAF6 are the oldest members, while TRAF1, 2, 3 and 5 arose later during vertebrate evolution [18,38]. Interestingly, the emergence of the TNFR family also occurred during this later period, suggesting that the last four TRAF proteins have a dedicated function that is directly linked to TNFR proteins. Given the observed major role of TRAF proteins during embryonic development of invertebrates and the major roles of TRAF4 orthologs in similar processes in vertebrates, TRAF4 may well be the TRAF family member with an ancestral morphogenetic function independent of TNFR [39].

## 4. TRAF4 Is Overexpressed in Cancers

**Figure 2.**
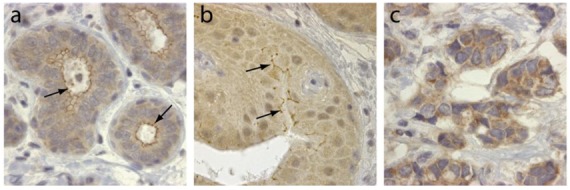
TRAF4 expression in normal breast and cancerous tissues. Immunohistochemistry of TRAF4 expression (brown) in normal breast (**a**), *in situ* carcinoma (**b**) and invasive carcinoma (**c**). Note that TRAF4 localization is modified from a predominantly membrane-associated presence in normal breast (a, arrows) to a mainly cytoplasmic presence in invasive tumor cells (c); *in situ* carcinomas represent an intermediate state in which TRAF4 remains associated focally with the membrane (b, arrows).

TRAF4 was initially identified in breast cancer from a differential screen between metastatic lymph nodes and benign fibrodenomas biopsies [3]. TRAF4 is located in the q11.2 region of the long arm of chromosome 17, close to the ErbB2 oncogene [3,4]. TRAF4 mRNA was shown to be overexpressed in approximately one-quarter of breast cancers as a consequence of gene amplification [40]. This was subsequently confirmed by comparative genomic hybridization (CGH) and cDNA microarrays [41-43]. Interestingly TRAF4 overexpression is not limited to breast cancers. Indeed, it has been found in 43% of 623 human tumor samples from lung, ovary, prostate and colon among others [44]. In this latter study, TRAF4 overexpression is caused by gene amplification in about one-third of the tumors. TRAF4 protein overexpression is therefore a common feature of human cancers. Accordingly, TRAF4 was identified in a meta-analysis of 40 independent microarray studies as one of the 67 genes whose overexpression is characteristic of carcinomas [45]. TRAF4 overexpression is always observed in cancer cells and never in stromal cells. Its subcellular localization is variable and is cytoplasmic and/or membrane-associated (Figure 2), but nuclear localization is also observed in about 20% of TRAF4-positive tumors [4,44]. Interestingly, the membrane-association of TRAF4 seems to be correlated with tumor cell polarity and differentiation (Figure 2). These studies strongly suggest that TRAF4 plays an important role in carcinogenesis. Interestingly, in breast cancers, TRAF4 amplification can occur independently of ErbB2 amplification suggesting that TRAF4 may be the target of a more centromeric amplicon than the one involving ErbB2 [40,44]. Collectively, these data suggest that TRAF4 is not only a marker of human carcinomas, but also a candidate oncogene.

In line with its elevated expression in tumors and its inclusion in the TRAF protein family, TRAF4 has been hypothesized to be involved in apoptosis. For instance, TRAF4 provides resistance to an apoptotic stimulus in HEK293 cells [46]. When treated with an agonistic anti-Fas antibody, HEK293 cells normally undergo apoptosis; however, TRAF4 overexpression in these cells strongly decreases induced-cell death, arguing for an anti-apoptotic function for TRAF4 [46]. Paradoxically, other studies have shown that TRAF4 might also exert pro-apoptotic functions. Indeed, TRAF4 is regulated by members of the p53 protein family, namely p53 and p63 [47,48]. Hence, TRAF4 can be up-regulated by a temperature sensitive p53 or by its overexpression, as well as by the stabilization of p53 in response to DNA damage. Moreover, induction of apoptosis by irradiation in human lymphocytes or in noise-exposed cochlea cells increases their expression of TRAF4 [49,50]. Interestingly, TRAF4 overexpression induces apoptosis and suppresses colony formation in cell lines of different origins, regardless of their p53 status. The apparent discrepancy among these results may reveal a cell-type specific role for TRAF4 in life and death decisions.

The elevated expression of TRAF4 in many carcinomas suggests that this protein might have an important role in cancer which could be linked to cell polarity or apoptosis regulation.

## 5. TRAF4 has Restricted Functions in Immunity

TRAF proteins interact with Toll/IL1R and TNFR receptor family members to induce immune and inflammatory responses through NF-κB and MAPK activation [6]. However, TRAF4 involvement in these mechanisms remains poorly understood. Indeed, TRAF4 activates or inhibits NF-κB pathway depending on the ligand/receptor pair; for instance, TRAF4 potentiates NF-κB activation triggered by glucocorticoid-induced TNFR (GITR) but inhibits LPS-induced NF-κB activation [51,52].

The role of TRAF4 in immunity was studied in TRAF4-deficient mice. TRAF4-deficient mice do not show defects in T and B lymphocytes, granulocytes, macrophages and dendritic cell differentiation [53]. Moreover, the function of neutrophils and T cells is unaffected. The only observed phenotype is reduced dendritic cell migration [53]. However, TRAF4-deficient mice have not been challenged for bacterial resistance or autoimmune diseases and a role for TRAF4 in these processes cannot be ruled out.

Recently, TRAF4 has been proposed as a key negative regulator of NOD2 (nucleotide-binding oligomerization domain containing 2) signaling [54]. NOD2 is an intracellular pattern recognition receptor which plays an important role in immunologic homeostasis [55]. The NOD2 gene is linked to inflammatory diseases such as Crohn's Disease and Blau syndrome. TRAF4 inhibits NOD2-induced NF-κB activation through direct binding to NOD2 and NOD2-induced bacterial killing [54].

Thus, TRAFs are key proteins in innate and adaptative immune signaling with positive and negative regulatory functions. TRAF4 seems to be a negative regulator of immunity in specific processes of innate immunity.

## 6. TRAF4 Regulates Reactive Oxygen Species (ROS) Production

ROS act as signaling molecules that mediate growth-related responses such as angiogenesis. As a result, abnormal ROS production is implicated in various diseases including atherosclerosis, cardiovascular problems and cancers [56,57]. The NADPH oxidase Nox2 is a multi-subunit complex comprising two membrane-bound subunits, gp91phox (Nox2) and p22phox, three cytoplasmic subunits, p47phox, p40phox and p67phox, and the small GTP-binding protein, Rac1/2 [58]. This complex generates superoxide by transferring electrons to molecular oxygen [57]. Superoxide is involved in bacterial killing, inflammation and endothelial activation. To better understand the molecular mechanisms of superoxide synthesis regulation, a two-hybrid screen using p47phox as a bait allowed the recovery of TRAF4 [59]. The C-terminal part of p47phox interacts with the TRAF domain of TRAF4. Coexpression of p47phox and TRAF4 induces JNK activation and ROS production whereas each alone does not appreciably affect these functions [59]. Acute response to TNF-α induces a rapid PKC-dependent p47phox phosphorylation [60]. This post-translational modification enhances p47phox-TRAF4 association and translocation to the cell membrane leading to NADPH oxidase activation and ROS generation. Moreover, ROS-induced ERK1/2 and p38MAPK activation requires both p47phox and TRAF4 [60]. In addition, TRAF4 and p47phox target nascent lamellar focal complexes of motile endothelial cells in association with the paxillin paralogue Hic-5 to initiate Rho GTPase signaling [61]. Downregulation of one of these proteins is sufficient to inhibit endothelial cell migration.

In a recent report, TRAF4 and p47phox were identified as binding partners for GPIb-IX-V and GPVI, two platelet glycoprotein receptors that respectively bind to Von Willebrand Factor (VWF) and collagen and initiate thrombosis [62]. These two receptors initiate signaling events leading to the activation of platelet integrin which mediates platelet aggregation. The cytoplasmic regions of GPIb-IX-V and GPVI interact with the TRAF domain of TRAF4 and with the SH3 domain of p47phox in a complex with Hic-5 [62]. Since the regulation of platelet adhesion receptors by redox changes remains poorly understood, this novel interaction could link these receptors to redox signaling pathways.

Together these studies indicate that TRAF4 forms part of a complex that regulates ROS production by the NADPH oxidase Nox2 and couples this regulation to cell migration.

## 7. TRAF4 and Cell Polarity

**Figure 3.**
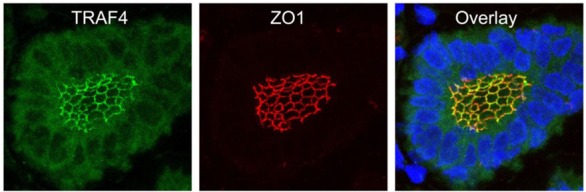
TRAF4 is localized at tight junctions (TJs) of normal human mammary epithelial cells. TRAF4 (green) and ZO1 (red) were co-labeled on paraffin embedded sections of a normal human breast duct. Images are confocal section projections showing the typical honeycomb-like shape of tight junctions. Nuclei were stained using Hoechst-33258 (blue).

TRAF4 has been detected in several cellular compartments: the plasma membrane, the cytoplasm and the nucleus [2,4,44]. When expressed in cells, TRAF4 is mainly found in the insoluble fractions of cell extracts [63]. Moreover, TRAF4 was shown to be associated with cytoskeleton- and membrane-containing fractions [59]. This membrane association is also observed in cells overexpressing TRAF4 where it accumulates at cell-cell contacts [64,65]. In *in vitro* polarized epithelial cells, TRAF4 localizes apically in cell-cell junctions, where it co-localizes with markers of tight junctions (TJ) such as occludin or Zonula Occludens-1 (ZO1) [65]. In normal human mammary samples, TRAF4 is found in TJ typical honeycomb patterns in epithelial cells (Figure 3). The association with TJ is highly dynamic as demonstrated by fluorescence recovery after photobleaching (FRAP) experiments, which show that TRAF4 shuttles between TJs and the cytoplasm. These data suggest that TRAF4 might be a signal transducer of intercellular junctions in normal epithelial cells. In contrast, in poorly differentiated epithelial cancer cells, the protein is either diffuse in the cytoplasm, or in cytoplasmic or nuclear foci, suggesting that this function is impaired (Figure 2) [65].

TJs are major cell-cell contact structures essential for the establishment and the maintenance of apico-basal polarity. A key regulator of cell polarity is TGF-β which is involved in the epithelial-mesenchymal transition (EMT), a process that allows cells to lose their polarity and cell-cell contact and acquire a mesenchymal phenotype. An intriguing link between the TRAF4 and TGF-β pathways came from the identification of TRAF4 as a potential interacting partner of Smurf1 and Smurf2 in two-hybrid screens [66-68]. Smurf (SMAD specific E3 ubiquitin protein ligase) proteins (Smurf1 and Smurf2) are related E3 ubiquitin ligases of the C2-WW-HECT class that regulate the TGFβ signaling pathway, motility, epithelial polarity and planar cell polarity [69,70]. *In vitro* assays confirmed that Smurf1 interacts with TRAF4 through 3 PXXY motifs located in the RING, zinc fingers and TRAF domains [67]. When ectopically expressed in cells, TRAF4 and Smurf1 co-localize in structures associated with the cell membrane [68]. Smurf1 was further shown to induce TRAF4 ubiquitination which in turn promoted TRAF4 26S proteasome-dependent degradation [67,68]. Thus Smurf1 can regulate TRAF4 protein levels through its E3-ligase activity.

Interesting data have come from the study of asymmetric cell division in *Drosophila*. In this organism, neuroblasts divide perpendicularly to the surface in the procephalic neurogenic region, and segregate the cell fate determinants Prospero (Pros) and Numb and their adaptor protein Miranda (Mira) into the future ganglion mother cells (GMCs) [71,72]. Interestingly DTRAF1, the ortholog of TRAF4, is asymmetrically localized to the apical cortex in mitotic neuroblasts [72]. This localization relies on Bazooka, the *Drosophila* ortholog of the polarity protein Par3 (Partitioning defective 3 homolog) with which DTRAF1 interacts, and depends on Eiger, the ortholog of TNF [72]. Thus DTRAF1 binds to Bazooka and acts downstream of Eiger in the Mira/Pros pathway during asymmetric division [73].

These studies suggest that mammalian TRAF4, located in the TJ of polarized epithelial cells and whose function in these structures might be linked to the TGF-β pathway, may be important in asymmetric division in specific cell types.

## 8. TRAF4 is Essential in Developmental Processes

TRAF4 function has been studied in different animal models. We summarize below the different findings obtained in each organism.

### Mouse

TRAF4 has been demonstrated to be essential in mice. TRAF4-deficient mice on a mixed 129/SvJ X C57BL/6 genetic background showed important defects of the upper respiratory tract [74]. On a pure 129/SvJ background this phenotype is more drastic and leads to 30% embryonic lethality [75]. Moreover, TRAF4-deficient survivors exhibit numerous less penetrant alterations, including dramatic malformations of the trachea and axial skeleton (ribs, sternum, tail), and defects in neural tube closure giving rise to mild spina bifida phenotypes [75]. Interestingly, these neural tube and skeletal defects are similar to those found in MEKK4-deficient mice, a MAP3K protein kinase of the STE11 family involved in the JNK/p38 pathway [76,77]. Some of these phenotypes might be linked to neural crest defects. Indeed, a TRAF4 mutant allele called msp2 (modifier of Sox10 expression pattern 2) alters neural crest patterning [78]. As discussed, defects in dendritic cells migration has also been reported in TRAF4-deficient mice which otherwise exhibit normal immune responses [53]. These null-phenotypes coincide with the embryonic expression pattern of murine TRAF4 [79]. Indeed, TRAF4 expression is highly regulated during development. It is widely expressed in various developing organs, with the highest levels observed during ontogenesis of the central (CNS) and peripheral (PNS) nervous systems, and in the nervous tissues of sensory organs. Thus, TRAF4 is a key molecule in diverse ontogenic processes, particularly in the nervous system [79]. In line with its potential role in the nervous system, TRAF4 expression was reported to be reduced in the temporal cortex of schizophrenic patients [80]. In adult mice, constitutive basal expression of TRAF4 is observed in most tissues, suggesting a general biological function for this protein [79]. A similar broad expression pattern is observed in human adult tissues [32,81]. Indeed, the promoter region of the TRAF4 gene does not have a consensus TATA box and contains a relatively weak Kozak sequence, two characteristics of ubiquitously expressed genes. Nonetheless, in mice, the hippocampus, the olfactive bulb, and the Purkinje cells of the cerebellum show strong TRAF4 expression, indicating that TRAF4 might also exert additional tissue-specific function(s) [79,82].

### Zebrafish

In zebrafish, two TRAF4 orthologs have been found, TRAF4a and TRAF4b [82]. Many mammalian genes have two zebrafish orthologs due to genome duplication during the evolution of these ray-finned fish [83]. Both TRAF4a and TRAF4b start to be expressed very early during embryogenesis. However, TRAF4b is ubiquitously and weakly expressed at all stages, while TRAF4a shows a highly restricted expression pattern mainly in sensorial and neural cells, and in the somites of embryos. Together, TRAF4a and TRAF4b have similar expression pattern as their mammalian counterpart [82].

### Xenopus

The TGF-β pathway is a major signaling pathway during early developmental events in the *Xenopus* embryo. The E3 ubiquitin ligase Smurf1 negatively regulates TGF-β signaling, therefore, targets or partners of Smurf1 were proposed to participate in TGF-β signaling during embryonic processes [68]. TRAF4 was found in a yeast two-hybrid screen for Smurf1-interacting proteins. In the *Xenopus* embryo, two TRAF4 paralogs have been cloned, TRAF4a and TRAF4b, both of which are expressed within the neural plate and neural crest and are essential for neural crest development and normal neural plate folding [68]. TRAF4 is a positive effector of BMP (Bone morphogenetic protein) and Nodal, two TGF-β superfamily ligands. Indeed, endogenous TRAF4 is needed for the response of animal caps to these pathways. In contrast, TRAF4 has negligible effects on the Wnt (Wingless) and FGF (fibroblast growth factor) signaling, two other crucial pathways for neural crest specification.

### Drosophila

The TRAF4 ortholog in *Drosophila* is DTRAF1 [84,85]. Homozygous flies carrying a null allele of DTRAF1 fail to develop to the pupal stage [86,87]. DTRAF1 seems to be necessary as early as the gastrulation stage as DTRAF1-deficient embryos show delays in ventral furrow formation [86]. Mutant larvae contain smaller imaginal discs, especially eye discs, form few axonal bundles and fail to defasciculate in the brain hemisphere. Heterozygous embryos show a dramatically enhanced thorax closure defect due to dominant negative expression of JNK (c-Jun N-terminal kinase) showing the importance of gene dosage [87]. Flies with a hypomorphic DTRAF1 mutation due to homozygous P-element insertion show supernumerary dorsal bristles, normally a typical structure of the fly peripheral nervous system [88]. Altogether, these results indicate that DTRAF1 is essential for thorax closure, development of imaginal eye discs and the formation of photosensory neuronal array in the brain hemisphere.

The reaper (rpr) gene is a key regulator of apoptosis *during Drosophila* embryogenesis [89]. DTRAF1 induces cell death through apoptosis signal-regulating kinase (ASK1) and JNK activation [88]. *Drosophila* inhibitor-of-apoptosis protein 1 (DIAP1) appears to negatively regulate apoptosis by triggering DTRAF1 proteasome-mediated degradation. Reaper, through DIAP1 inhibition, induces the stabilization of DTRAF1 and consequently JNK-mediated apoptosis. Misshapen (msn), an SPS1 (sporulation-specific 1) subfamily Ste20-related kinase, was shown to be an upstream activator of the JNK pathway required for dorsal closure. Msn directly binds to the TRAF domain of DTRAF1 and thereby potentiates DTRAF1-induced JNK activation [85].

Eiger, the *Drosophila* ortholog of TNF, is known to activate JNK and trigger apoptosis. An essential component of the Eiger-JNK pathway is dTAB2, the *Drosophila* ortholog of TAB2/3 (TGF-beta activated kinase 1(TAK1) binding protein 2/3). dTAB2 links DTRAF1 to the JNK kinase kinase, dTAK1, to form a triple complex [90]. This complex is recruited to the membrane through the interaction with DTRAF1 and the Eiger receptor, Wengen. This complex has an adaptor function which enables the presumptive JNKKKK Misshapen to activate dTAK1, which in turn advances the Eiger signal to the JNKK hemipterous and its substrate JNK. These data show that Eiger uses the dTAB2-DTRAF1-dTAK1 module to induce the JNK signaling pathway in *Drosophila* [90].

In *Drosophila*, the Toll signaling pathway regulates biological mechanisms such as dorso-ventral polarity, larval hematopoiesis and innate/adaptive immunity. Upon ligand binding to the Toll receptor, Pelle, a serine/threonine protein kinase homologous to the mammalian kinase IRAK (interleukin-1 receptor-associated kinase), becomes activated and phosphorylates itself and several other substrates. This leads to the activation and nuclear translocation of the *Drosophila* counterpart of mammalian NF-κB, Dorsal. DTRAF1 interacts with the regulatory N-terminal domain of Pelle [91]. Interestingly, coexpression of DTRAF1 and Pelle induces significant NF-κB activation whereas each alone does not. These data suggest that DTRAF1 is a component of the Toll pathway in *Drosophila*.

Altogether, these results show that the *Drosophila* ortholog of TRAF4, DTRAF1, is a key element of the JNK signaling pathway downstream of the *Drosophila* TNF pathway which is involved in several developmental processes. Interestingly, TRAF4 interacts with MEKK4 in human cells to activate JNK [92]. This suggests that TRAF4 may be involved in the JNK pathway together with MEKK4 in humans.

The study of TRAF4 in different model organisms has shed light on the crucial role of this protein in several physiological processes, in particular during embryonic development [39].

## 9. Conclusions

TRAF4 is overexpressed in many different types of carcinomas. However, the precise implication of this protein in cancer remains poorly understood. A clearer picture of its physiological function has, however, appeared. The study of TRAF4 in different organisms has revealed that it is involved in different morphogenic processes. Its function seems to be primordial with links to cell polarity and different pathways such as ROS production and TGF-β signaling.
